# Effects of Tween 20 addition on electrokinetic transport in a polydimethylsiloxane microchannel

**DOI:** 10.1002/elps.202400024

**Published:** 2024-03-21

**Authors:** Seyed Mojtaba Tabarhoseini, Joseph Bentor, Walter Johnson, Tzuen‐Rong Tzeng, Xiangchun Xuan

**Affiliations:** ^1^ Department of Mechanical Engineering Clemson University Clemson South Carolina USA; ^2^ Department of Biological Sciences Clemson University Clemson South Carolina USA

**Keywords:** electrokinetic, electroosmosis, electrophoresis, microfluidics, surfactant

## Abstract

Tween 20 is frequently added to particle suspensions for reducing the particle–wall adhesion and particle–particle aggregation in microfluidic devices. However, the influences of Tween 20 on the fluid and particle behaviors have been largely ignored. We present in this work the first experimental study of the effects of Tween 20 addition on the electrokinetic transport of fluids and particles in a polydimethylsiloxane microchannel. We find that adding 0.1% v/v Tween 20 to a buffer solution can significantly reduce the electroosmotic mobility as well as the electrokinetic and electrophoretic mobilities of polystyrene particles and yeast cells. Further increasing the Tween 20 concentration within the range typically used in microfluidic applications continues reducing these mobility values, but at a smaller rate. Our finding suggests that Tween 20 should be used with care in electrokinetic microdevices when the flow rate or particle/cell throughput is an important parameter.

Tween 20 is a nonionic detergent frequently used as a surfactant in microfluidic devices to reduce the particle–wall adhesion and particle–particle aggregation. These functions have been demonstrated in both pressure [1, 2, 3] and electric field‐driven [4, 5, 6] flows of Newtonian [7, 8, 9] and non‐Newtonian [10, 11, 12] fluids through polydimethylsiloxane (PDMS) microchannels. However, the effects of Tween 20 addition on the fluid flow and particle motion have been largely ignored. Our recent study indicates that the addition of Tween 20 at the concentration commonly used in microfluidic applications reduces the viscosity and stabilizes the extensional flow of shear‐thinning fluids in a cavity microchannel. It, however, has no significant impact on Newtonian water or Boger fluid [13]. We perform in this work an experimental investigation of the effects of Tween 20 addition on the electrokinetic transport of fluids and particles in a PDMS microchannel. We examine how the variation of Tween 20 concentration in a buffer solution affects the electroosmotic fluid flow and the electrokinetic/electrophoretic motions of particles and cells.

The PDMS microchannel was fabricated using the standard soft‐lithography technique [14]. It is 1 cm long and has a rectangular cross‐section of 50 µm wide and 35 µm deep. Spherical polystyrene particles (Sigma‐Aldrich) of 5 µm diameter were resuspended in 1 mM phosphate buffer to a final concentration of around 10^6^ particles per mL. Yeast cells (*Saccharomyces cerevisiae*) were also tested under similar conditions. Both the particle and cell suspensions were mixed with Tween 20 (Fisher Scientific) at the concentration ranging from 0.1% to 0.5% v/v. Our previous measurements indicate that the addition of 0.5% v/v Tween 20 does not cause a noticeable change to the viscosity of water [13]. We therefore assume that the prepared buffer solutions have a similar viscosity to water, regardless of the concentration of Tween 20 therein.

The particle or cell suspension was driven through the microchannel by a high‐voltage DC power supply (Glassman high voltage). The electric field strength was varied from 100 to 300 V/cm. No higher electric fields were tested to minimize the effects of Joule heating [15] and any other nonlinear electrokinetic phenomena such as nonlinear electrophoresis [16]. The electrokinetic motion of particles or cells was recorded in the middle of the microchannel using an inverted microscope imaging system (Nikon Eclipse, TE2000U, Nikon Instruments) with a CCD camera (Nikon DS‐Qi1Mc). Its velocity, $V_{ek}$, is given by, $V_{ek}=V_{eo}+V_{ep}$, where $V_{eo}$ is the electroosmotic fluid velocity, and $V_{ep}$ is the electrophoretic particle or cell velocity. The value of $V_{ek}$ was measured using the particle‐tracking velocimetry, where at least five particles or cells traveling along the channel centerline were considered in the measurement. The value of $V_{eo}$ was measured using the electric current monitoring technique [17]. Each of these measurements was repeated at least twice to verify the consistency. The electrophoretic velocity, $V_{ep}$, was determined from $V_{ep}=V_{ek}-V_{eo}$. It is important to note that all our measurements were conducted in freshly prepared microchannels as the surface charge of PDMS has been reported to change over time [18].

Figure 1 shows the experimental data for the electroosmotic velocity, $V_{eo}$, of buffer solutions with the concentration of Tween 20 ranging from 0 (i.e., surfactant‐free) to 0.5% v/v under different DC electric fields. Two clear trends are observed from the line plots. One is the (nearly) linear dependence of $V_{eo}$ on electric field, E, across all fluids, indicating an insignificant disturbance of surfactant addition to the linear regime of electroosmosis under our experimental conditions. We therefore can determine the electroosmotic mobility, $\mu_{eo}=V_{eo}/E\;$, from the slope of the linear trend line to the experimental data in Figure 1 for each tested fluid. The other trend is the decrease of $V_{eo}$ with the increasing concentration of Tween 20, which may arise from the charge screening effect of this nonionic surfactant because of its adsorption on the microchannel walls [19]. The effect of surfactant addition on the value of $\mu_{eo}$ will be presented later.

**FIGURE 1:**
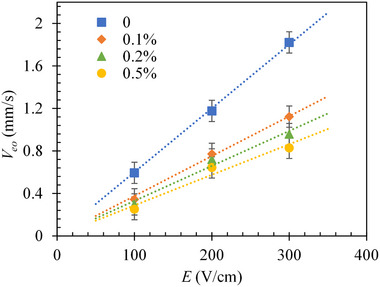
Experimentally measured electroosmotic velocity, $V_{eo}$, in buffer solutions mixed with varying concentrations of Tween 20 under a range of DC electric fields, E. The dashed lines are each a linear fit to the experimental data (symbols with error bars).

Figure 2A shows the experimentally measured electrokinetic velocity, $V_{ek}$, of polystyrene particles in buffer solutions with varying Tween 20 concentrations. Two similar trends to those in Figure 1 are again observed here. The linear increase of $V_{ek}$ with increasing electric field in all prepared fluids further confirms the linear regime of electrokinetics under our experimental conditions [20]. This trend enables us to determine the electrokinetic mobility, $\mu_{ek}=V_{ek}/E\;$, from the slope of the linear trend line to the experimental data in Figure 2A for each tested fluid, which will be presented shortly along with $\mu_{eo}$. The decreasing trend of $V_{ek}$ with the increasing concentration of Tween 20 is attributed to its suppression effect on the surface charge of both the particles and microchannel walls [19]. Figure 2B shows the electrophoretic particle velocity, $V_{ep}$, determined from the experimentally measured $V_{eo}$ (Figure 1) and $V_{ek}$ (Figure 2A). It is not surprising to see that $V_{ep}<0$ in all the prepared fluids because the electrophoretic motion of particles, which are intrinsically negatively charged [21], is against the direction of electric field. The obtained linear electroosmosis and linear electrokinetics together lead to linear electrophoresis, which is valid under small electric fields in the absence of nonlinear electrophoresis [22, 23, 24].

**FIGURE 2:**
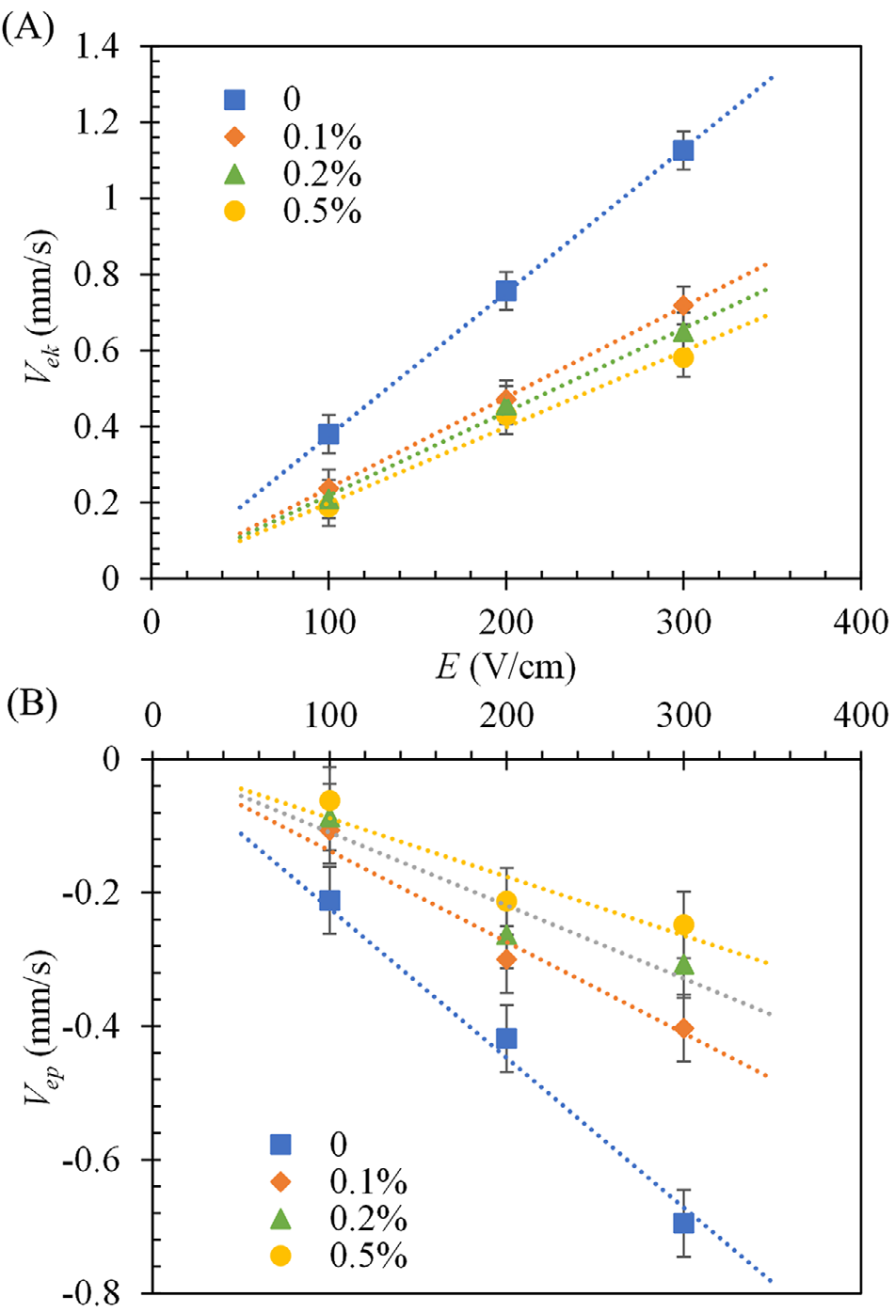
Effects of Tween 20 addition on (A) the experimentally measured electrokinetic velocity, $V_{ek}$ (positive as it is along the direction of electric field), and (B) experimentally determined electrophoretic velocity, $V_{ep}$ (negative as it is against the direction of electric field), of polystyrene particles under a range of DC electric fields, E. The dashed lines are each a linear fit to the experimental data (symbols with error bars).

Figure 3A presents the effects of Tween 20 concentration on the electroosmotic mobility, $\mu_{eo}$, electrokinetic mobility, $\mu_{ek}$, and electrophoretic mobility, $\mu_{ep}$, of polystyrene particles, which were obtained from the slopes of the linear trend lines in Figures 1 and 2, respectively, as stated earlier. A significant observation is the quick decline in each of these mobilities when the concentration of Tween 20 is increased from 0% (i.e., surfactant‐free) to 0.1%. This downward trend, however, becomes less pronounced for all three mobilities as the Tween 20 concentration is further increased. Such a pattern implies that these mobility values may level off at even higher Tween 20 concentrations, where a certain physical or chemical equilibrium condition, such as the saturated adsorption of Tween 20 molecules onto the channel walls and particle surfaces [19], is perhaps achieved. Figure 3A also shows the effects of Tween 20 concentration on $\mu_{ek}$ and $\mu_{ep}$ of yeast cells, each of which follows a similar curve to that for polystyrene particles. Figure 3B plots the three mobilities for polystyrene particles and yeast cells that are each normalized by the corresponding value in the surfactant‐free buffer solution. The curves of $\mu_{ek}$ and $\mu_{ep}$ sandwich that of $\mu_{eo}$ for both the particles and cells over the range of Tween 20 concentrations. However, the gaps among the three curves for the cells are overall larger than those for the particles, especially at the lower concentrations of Tween 20. This discrepancy may arise from the different physiochemical compositions of the particles and cells. It is further noted that all the normalized mobilities drop to 0.5 (±0.1) with no more than 20% deviations at 0.5% Tween 20. The former number is significant and needs to be taken into consideration for electrokinetic microdevices where the flow rate or particle/cell throughput is an important parameter. Figure 3C replots the three mobilities in a log–log space, each of which exhibits a negative‐power‐law dependence on Tween 20 concentration. More data points, especially at higher Tween 20 concentrations, are needed to validate this relationship.

**FIGURE 3:**
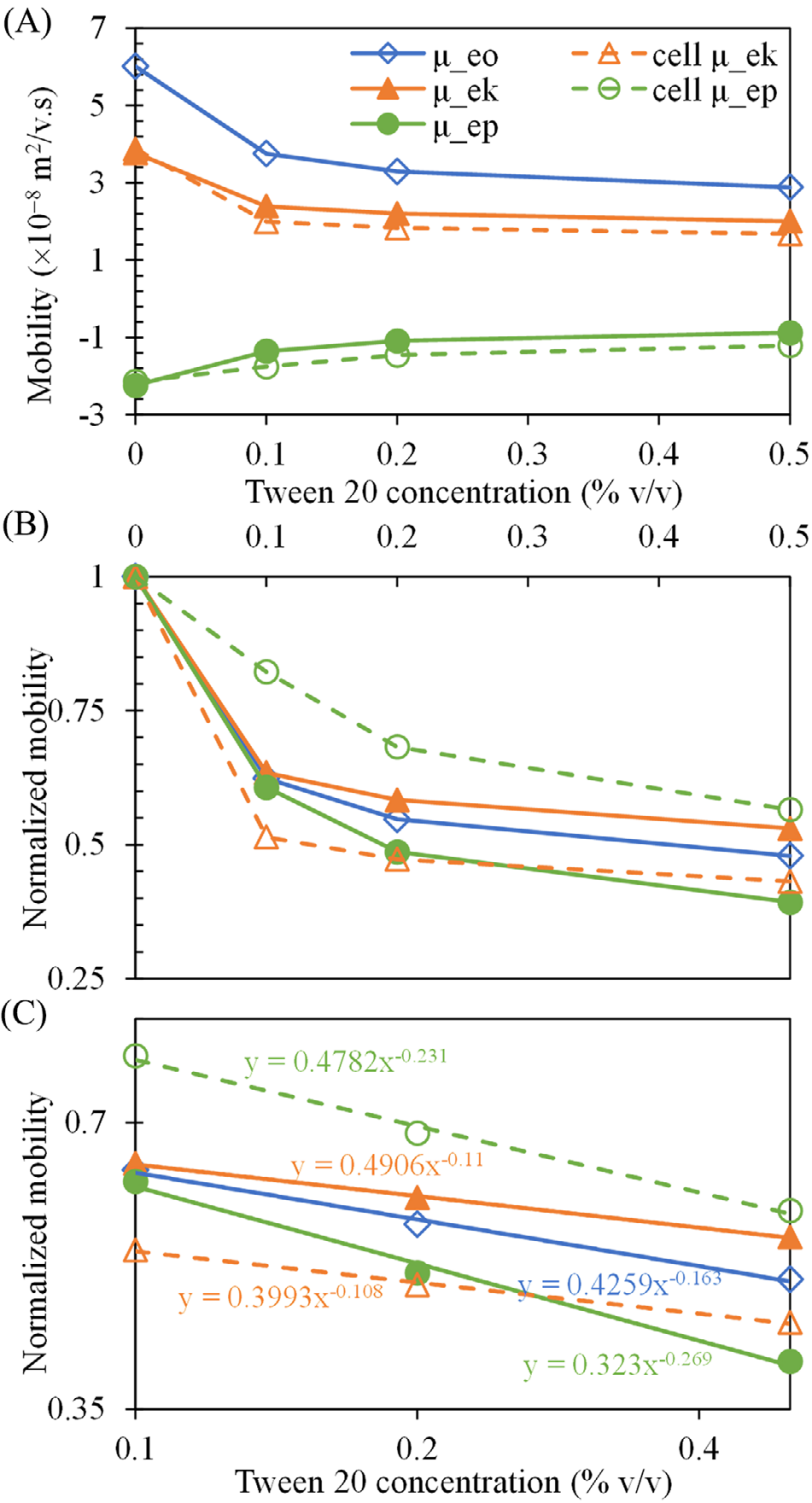
Effects of Tween 20 concentration on (A) the dimensional and (B) normalized (by the corresponding value at zero concentration of Tween 20) electroosmotic mobility, $\mu_{eo}$, electrokinetic mobility, $\mu_{ek}$, and electrophoretic mobility, $\mu_{ep}$, of both polystyrene particles (filled symbols with solid lines) and yeast cells (hollow symbols with dashed lines). All the lines are used to guide the eyes only. Part (C) shows the normalized mobilities versus Tween 20 concentration in a log–log space, where the lines are each a power trend line best fitted to the data with the equation displayed on the chart.

In summary, we have reported the first experimental study of the effects of Tween 20 addition on the electrokinetic transport of fluids and particles in a PDMS microchannel. We find that the introduction of 0.1% v/v Tween 20 into the buffer solution results in a notable reduction in both the electroosmotic mobility and the electrokinetic/electrophoretic mobilities of polystyrene particles and yeast cells. Such a decreasing trend continues with the increase of Tween 20 concentration for all mobility values but appears to level off when the Tween 20 concentration goes beyond the range typically used in microfluidic applications. This phenomenon may suggest the existence of a physical or chemical equilibrium between the surfactant molecules and the channel walls or particles/cell surfaces. In future work, we will investigate how the addition of Tween 20 affects the electrokinetic transport of fluids and particles in non‐Newtonian polymer solutions [25, 26].

## CONFLICT OF INTEREST STATEMENT

The authors have declared no conflicts of interest.

## Data Availability

The data that support the findings of this study are available from the corresponding author upon reasonable request.
